# Tactical and surgical techniques issues in the surgical treatment of incisional hernias

**Published:** 2014-09-25

**Authors:** AG Gangură, RŞ Palade

**Affiliations:** * Surgery Clinic 2, University Emergency Hospital Bucharest; ** Surgery Clinic 1, University Emergency Hospital Bucharest

**Keywords:** surgical techniques, incisional hernias

## Abstract

Abstract

Within five years, between 2006 and 2011, a total of 368 incisional hernias have been operated in the Surgery Clinic 1, University Emergency Hospital Bucharest. The study followed the morphological and biological parameters, associated pathology, tactics and surgical technique used and postoperative morbidity. The average age of patients was 61.75 years, female sex was predominant (81.25%), and incisional hernias were large and giant in a percentage of 73.37%. Locations were predominantly median (83.42%). Recurrent incisional hernias and multiple relapsed hernias represented 25.54%. Associated pathology was dominated by obesity (51,09%) and cardiovascular disease (37,77%). We have used both methods of tissue procedures (22.83%), and the prosthetic procedures (77.17%). Prosthetic techniques, retro muscle fitting mesh in the rectus abdominis muscle sheath (Rives-Stoppa technique), fitting ov er the fascia and tissue replacement techniques were performed. Immediate postoperative morbidity was represented by seroma (14.13%), prolonged postoperative ileus (8.69%), prolonged hematic drainage (6.52%), and hematoma (1.9%). Late postoperative morbidity was given by granulomas (5.7%) and recurrence of incisional hernias (4.34%). Good and very good results were obtained in the 89.96% of the operated cases.

## Introduction

According to the diversity of clinical manifestations and pathological form, incisional hernias required individualized surgical tactics and techniques in order to obtain an optimal and lasting restoration of the muscular defect.

 Surgical tactic requires: 

 -a good knowledge of the associated pathology, in order to be able to correct it preoperatively;

 -an evaluation of the morphological characteristics of the defect; 

 -a rational attitude towards the risks and benefits of the surgical cure, which will ultimately determine the correct choice of the practiced surgical technique.

 Thereby, we are followers of the surgical cure of incisional hernias by prosthetic procedures. We believe the procedure envisaged by Jean Rives in 1973, then developed, with small changes, by Rene Stoppa is the safest and with superior benefits to the patient [**1**].

 However, depending on the individual situation (emergency or associated surgical pathology), we approached the following surgical techniques: tissue procedures, onlay aponeurosis mounted mesh, or plastic substitution, with good and very good results. 

 We present a retrospective study, which includes a period of five years (2006-2011), during which we analyzed this pathology and for which we used different surgical techniques adapted to each case.

## Material and method 

During 2006 and 2011, were operated 368 incisional hernias, both, scheduled intervention (332 cases - 90,22%) and emergency (36 cases - 9,78%).

 The average age of the patients was 61.75 years, with the extreme limits from 21 to 86.

**Table 1:** Age distribution of patients with incisional hernias

Age group	Number of patients	Percent
21 - 30 years old	4	1,09%
31 - 40 years old	6	1,63%
41 - 50 years old	44	11,96%
51 - 60 years old	94	25,54%
61 - 70 years old	132	35,87%
71 - 80 years old	82	22,28%
81 - 90 years old	6	1,63%

 The incidence of incisional hernias according to age, sixth and seventh decades followed by eight and five decades, has been the most affected. There have been 358 patients (97,28%) over 40 years. Incisional hernias occurred mainly after 40 years with the onset of trophic tissue involution process. Therefore, the age factor should be considered an important element in the development of this disease.

 The time since the last surgery or the one generating a muscular defect or a corrective surgery was on average of 14.8 months, with a range between 2 and 72 months.

 A different aspect, in terms of surgical approach, is the emergency intervention. In these cases, the surgical urgency resolution, which is typically incarceration, strangulation, obstruction or perforation of a hollow organ with local or diffuse peritonitis, takes precedence, and the treatment of the muscular defect passes in the background.

 Most patients were females, 299 cases (81.25%).

**Fig. 1:**
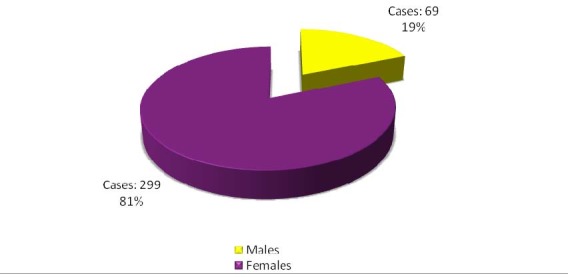
Gender distribution of patients with operated incisional hernias

 We confronted with many cases of large and giant incisional hernias: 270 cases - 73,37% (**Table 2**).

 Regarding location, we divided the study group in: median incisional hernias (307 cases - 83.42%) and other sites: subcostal - 31 cases (8,42%), right iliac fossa (post-appendectomy) - 16 cases (4,35%), pararectal - 8 patients (2,17%), in flanks - 4 cases (1,09%) and parastomal - 2 cases (0,54%).

 There were 94 cases (25.54%) of multiple recurrences specific to incisional hernias, meaning more than a quarter of the patients.

 Associated pathology was represented mainly by obesity - 188 cases (51.09%), cardiovascular diseases -139 cases (37.77%), consumptive diseases - 44 cases (11.95%).

 These types of associated pathologies often co-existed and have mutually intensified, causing a complex pathological picture that requires a specialized interdisciplinary collaboration to ensure a safely surgical treatment and good postoperative recovery.

**Table 2:** Classification according to muscular defect sizes of operated incisional hernias

Size	Number	Percent
Small (0-5cm)	3	0,82%
Medium (5-10cm)	95	25,82%
Large (10-15cm)	186	50,54%
Giant (over 15cm)	84	22,83%

**Table 3:** Associated pathology in patients with postoperative incisional hernias

Associated pathology	Number	Percent
No associated pathology	97	26,35%
Obesity	188	51,09%
Cardiovascular diseases	139	37,77%
Malignancies, Liver cirrhosis	44	11,95%
Diabetes Mellitus	35	9,51%
Pulmonary	20	5,43%
Genital	14	3,80%
Peritonitis	11	2,98%
Intestinal obstruction	8	2,17%

 Surgery was practiced in emergency for acute complications of incisional hernias, in 9.78% of cases. In the remaining patients, the surgery was scheduled (90.22%). The surgical techniques used are presented in Table 4.

**Table 4:** Surgical techniques used in emergency interventions and in the scheduled ones

Technique	Scheduled operations	Surgical emergency	Total
Tissue suture	70 (83,33%)	14 (16,67%)	84
Onlay prosthetic procedure	183 (91,96%)	16 (8,04%)	199
Stoppa procedure	71 (92,21%)	6 (7,79%)	77
Prosthetic tissue substitution	8 (100%)	0	8
Total	332 (90,22%)	36 (9,78%)	368

 General anesthesia with oro-tracheal intubation was used in most of the cases. Spinal and epidural anesthesia was also practiced. In most of the cases, postoperative pain control was made by mounting an epidural catheter before anesthesia.

 A prophylactic antibiotic, usually a combination of cephalosporin and aminoglycoside, was administered intraoperatively. Antibiotic therapy was continued postoperatively for 5-7 days.

 Omentectomy was the main associated surgical procedure for incisional hernias surgery - in 6.25% of the cases.

 We preferred to use aspiration drainage with dual role, for drain fluid collection and to abolish, by negative pressure, the remaining spaces resulting from extensive dissection required by the size of the hernia sac.

**Table 5:** Type of drainage

Drainage	Number	Percent
Above aponeurosis	273	74,18%
Mixed / above and under aponeurosis	57	15,49%
Under aponeurosis	10	2,72%
No drainage	28	7,61%

 In case of tissue suture technique and prosthetic onlay procedures, we used an aspiration drainage onlay the aponeurosis, and the tube was externalized through declive skin incision.

 In the case of mounting a retromuscular prosthesis (Rives-Stoppa procedure), the drainage was mixed, both behind the muscle and above aponeurosis.

 Drainage was not used when the aponeurotic defect was small and intraoperative hemostasis was achieved in a very good condition.

 Drains were maintained as long as they were productive, for 7-10 days postoperatively.

 Statistical analysis showed that the incarceration of the incisional hernias was one of the most common complications which occurred at older ages (**Table 6**).

**Table 6:** The average age of patients with incisional hernias non-incarcerated and incarcerated

Incisional hernia	Number of cases	Average age	Minimum	Maximum
Non-incarcerated	197	60,17	58,56	61,77
Incarcerated	171	63,56	61,97	65,15

## Results 

Immediate postoperative morbidity was represented by:

 • Seroma - 52 cases (14.13%), 

 • Prolonged postoperative ileus - 32 cases (8.69%), 

 • Prolonged hematic drainage - 24 cases (6.52%), 

 • Relapses of eventrations -19 cases (5.16%) 

 • Septic wound complications - 11 cases (2.98%), 

 • Haematoma -7 cases (1.9%), 

 • Thrombophlebitis of the lower limbs - 6 cases (1.63%), 

 • Minimum parietal necrosis, especially after the excision of skin and fatty tissue- 3 patients (0.81%).

 We have been forced to perform an early reintervention in seven cases for the evacuation of hematoma. 

 There have been no serious cardio-respiratory complications in the postoperative period. 

 Late postoperative morbidity was represented by granulomas - 21 cases (5.7%). 

 There were two deaths (0.5%). Causes of death were due to post-operative acute myocardial infarction in a patient of 76 years old and massive pulmonary embolism, in a 68-year-old patient with obesity.

 The average length of hospitalization was of eight days in our study.

 There were good and very good results in 331 cases (89.94%).

## Discussions

In literature, the incidence of postoperative eventrations is different:

 • primary, arising after laparotomy, ranges to 2-11% and if there are any postoperative complications, up to 23%;

 • recurrent incisional hernias in patients with prior tissue procedures up to 63% [**2-5**];

 • in prosthetic procedures, the relapse decreases to 2-36% [**6-8**];

 It is also known that eventration is the most common postoperative complication of abdominal surgery [**9**].

 What should be remembered is that the latent infections are commonly encountered in the female genitalia and pelvis, which explains the high frequency of eventrations at this level.

 In addition to infections, another important etiologic factor is aponeurosis damage by improper sutures [**11**].

 In our study, the time since the last surgery, which favored the emergence of incisional hernia, until the corrective intervention, averaged for 14,8 months. It is certain that the moment of musculo-aponeurotic fault precedes presentation to the doctor and the extent of complications is directly proportional to the time passed between the two moments mentioned [**10**].

 Obesity found in 51.09% of the patients with incisional hernias can be incriminated in the etiology for at least two reasons. Abdominal lipomatous tissue develops and diminishes the resistance of musculo-aponeurotic structures, and the lack of exercise, physical work of the individual, with poorly developed musculo-aponeurotic structures, leads to obesity.

 We believe that these two theories are summed and condition each other.

 Respiratory disorders accompanied most of the postoperative incisional hernias. They were an etiologic factor, both in the reduced amount of O2 provided to the scarring tissue and by the condition of “chronic cough", which creates high intra abdominal pressure repeatedly, especially immediately after surgery.

 The septic factor had a great importance among the etiologic factors in 32% of the studied cases.

 A significant percentage is represented by multiple recurrent incisional hernias - 25.54%. In most of these cases, the first operations were made by using tissue suture or onlay prosthetic repairing techniques. 

 In 104 patients (28.26%) with incisional hernias, as underlying disease, collagen diseases (hernia, varicose veins, flat feet, etc.) were encountered. In these patients, the incisional hernia relapse rate was of 62.5%.

 What should be noted is that 34% of postoperative incisional hernias occurred after cholecystectomy trough midline or Kocher incision.

 Surgical cure of umbilical hernia was followed by incisional hernia in 19% of cases. Obesity was frequently found in these cases as an underlying disease.

 Smoking was reported in 64.3% of males and 16.3% among women.

 Depending on the area of the parietal defect location and on the incision type, the following attitudes have been outlined: 

 - incisional hernias of median line received parietal prosthesis by using classical techniques; 

 - incisional hernias in other sites have raised particular problems of the parietal prosthesis without abandoning the classical general principles of mounting the prosthesis.

 The surgical treatment was individualized by taking into account the specifics of each case, in relation to the volume of the parietal defect, location, the trophicity of the structures, the presence or absence of the septic factor, associated pathology and scheduled or emergency nature of the intervention [**12**].

 The mesh prosthesis surgery of parietal defect was performed in 284 patients (77.17%) representing more than ¾ of all the cases.

 Parietal prosthesis technique is still a discussed and controversial issue. We believe that the Rives-Stoppa procedure, meaning the placing of prosthesis into the sheath of rectus abdominis muscle, is the most logical and fair manner for a safe and rapid integration of the prosthetic material in the parietal structure, and this procedure is preferred by most surgeons (golden standard) [**13-15**] .

 A faster and more convenient manner proved to be the placement of prosthesis onlay the aponeurosis. A method prone to complications, most commonly long and debilitating, solved only by the extraction of the prosthesis is preferred and recommended by some authors.

 During the intervention, the lysis of adhesions was imposed in 84% of cases. Sporadic damage of the intestinal loops has been present in around 15% of the cases, which were treated with a simple suture.

 In the context of obese patients in which the incisional hernias were voluminous, we were advocates of fat and skin excision that facilitated the approach of parietal defect and aesthetically correct the parietal deformity.

 Excision of skin and fatty tissue was associated with surgical procedures of incisional hernias in 26 cases (7.07%). It offered clear advantages for a more accurate surgical technique:

 - Widely exhibited muscular structures;

 - Allowed aponeurotic fixation of the prosthesis away from the edges (minimum 5 cm);

 - Decreased amount of abdominal fat, which had better postoperative results, both aesthetically and functionally.

 The interventions’ time averaged for 90 + / - 30 minutes.

 We have established anticoagulant therapy after surgery, in order to prevent thromboembolic disease by using subcutaneous low weight molecular heparins.

 Physical activity was gradually achieved, first in bed, passive and active, then by walking and physiotherapy. Socio-professional integration and the resumption of efforts were permitted after a minimum of three months after surgery.

 Elastic abdominal belt contention was used from the first day after surgery, also shortening the drainage time.

 The average length of hospitalization period was of eight days. In patients with serious underlying conditions and / or postoperative complications, the hospitalization was for 17 days.

 Choosing the best prosthesis, compatible with the structures, in compliance with all the technical details is a prerequisite for the success of the intervention. The presence of the septic factor, forced us to choose a macroporous prosthesis to prevent postoperative septic and inflammatory complications.
